# Association between Perceived Stress and Rhinitis-Related Quality of Life: A Multicenter, Cross-Sectional Study

**DOI:** 10.3390/jcm10163680

**Published:** 2021-08-19

**Authors:** Il Gyu Kong, Chae-Seo Rhee, Jung Woo Lee, Hyojin Yim, Min Jung Kim, Yunhee Choi, Doo Hee Han, on behalf of the Allergic Rhinitis Cohort Study Group

**Affiliations:** 1 Department of Otorhinolaryngology, Hospital Medicine Center, Seoul National University Hospital, Seoul 03080, Korea; yeah19@snuh.org; 2 Department of Otorhinolaryngology, Seoul National University College of Medicine, Seoul National University Hospital, Seoul 03080, Korea; csrhee@snu.ac.kr (C.-S.R.); jwooya83@naver.com (J.W.L.); byeolalae@naver.com (H.Y.); 3 Graduate School of Immunology, Seoul National University College of Medicine, Seoul 03080, Korea; 4 Institute of Allergy and Clinical Immunology, Seoul National University Biomedical Research Center, Seoul 03080, Korea; 5 Sensory Organ Research Institute, Seoul National University Biomedical Research Center, Seoul 03080, Korea; 6 Medical Research Collaborating Center, Seoul National University Hospital, Seoul 03080, Korea; kmj1094@naver.com (M.J.K.); yhchoi@snuh.org (Y.C.)

**Keywords:** allergic rhinitis, stress, disease-related quality of life, cross-sectional study, rhinoconjunctivitis quality of life questionnaire, stress management, Allergic Rhinitis Cohort

## Abstract

Allergic rhinitis (AR), a common chronic disease, impairs patients’ quality of life (QoL). This study aimed to determine the effect of perceived stress on disease-related QoL in AR patients. There were 741 patients from eight medical centers of the Allergic Rhinitis Cohort (ARCO) study. Data on sociodemographics, chronic conditions, AR severity, perceived stress level and Rhinoconjunctivitis QoL Questionnaire (RQLQ) results, and laboratory test findings were collected. The relationship between perceived stress and total RQLQ was analyzed using multiple linear regression. Potential confounding variables were adjusted. A high perceived stress level was associated with a high total RQLQ, which reflected worsening disease-related QoL. The high stress level was associated with an increased total RQLQ of 1.210 (95% confidence interval, 0.831–1.589; *p* < 0.0001) compared with the very low level. In the final model, the multiple regression-adjusted R^2^ for RQLQ in AR participants was 0.5279, and perceived stress levels contributed 4.08% in additional explanatory power to RQLQ in AR patients. In conclusion, perceived stress is a potentially modifiable risk factor for decreased disease-related QoL in patients with AR, which may be improved with stress management.

## 1. Introduction

Allergic rhinitis (AR) is one of the most common chronic diseases with a high global burden. It is characterized by an IgE-mediated immune reaction to allergens. In a European multicenter study [1], the age- and sex-standardized prevalence of AR in people aged 20 to 44 years ranged from 11.8% to 46.0%. In the International Study of Asthma and Allergy in Childhood (ISAAC) [2], AR prevalence increased from 8.5% in individuals aged 6 to 7 years to 14.6% in those aged 13 to 14 years. The 2010 Korea National Health and Nutrition Examination Survey [3] reported that the overall prevalence of AR based on laboratory tests was 16.2%.

Intranasal steroid therapy is the mainstay of treatment for persistent AR, and it is also the preferred treatment for moderate to severe AR. Allergen immunotherapy may be indicated in AR patients who have insufficient symptom control despite medication [4,5]. However, in clinical practice, symptom control is often insufficient despite treatment, leading to a decrease in the quality of life (QoL) of AR patients.

AR impairs the QoL of patients by affecting their sleep quality and their performance at work, school, or during leisure activities. AR is frequently associated with comorbidities such as asthma and conjunctivitis. Therefore, the Allergic Rhinitis and its Impact on Asthma (ARIA) guidelines have proposed criteria for AR severity, which consider the effects of AR on QoL [6]. Moreover, disease-specific QoL measurements are the best indicators of AR burden. Measuring QoL ensures that important symptoms are not overlooked and presents symptoms as an objective score, thereby allowing a comprehensive comparison of disease control status.

Stress can influence immune response, aggravate inflammation, delay wound healing, and increase susceptibility to infection [7]. Many studies have been conducted to demonstrate the correlation between stress and various allergic diseases, and it has been proven that the occurrence of allergic reactions increases when the environment is stressful [8,9,10,11]. Studies have also suggested that emotional status could affect the allergic response in various diseases [12,13,14]. Individuals with persistent emotional stress have been found to have frequent allergy flares [15]. However, there are limited studies on the association between stress and disease-related QoL in AR patients, and the relationship between these variables remains unclear.

We have already demonstrated in a previous study [16] involving the Allergic Rhinitis Cohort (ARCO) that stress is a risk factor for pediatric AR. In this study, it was demonstrated that children without enough play time were more likely to have AR than those with enough playtime [16]. Therefore, we hypothesized that, as observed in other chronic inflammatory diseases, perceived stress levels in AR patients might be related to disease-related QoL in AR patients. The purpose of this study was to evaluate the association between perceived stress and disease-related QoL in Korean adult patients with AR using the data from the ARCO study.

## 2. Materials and Methods

### 2.1. Study Population and Data Collection

This study used data from the ARCO study. The ARCO study is a national project designed to comprehensively investigate the clinical features of AR, comorbidities, and socioenvironmental factors of AR patients. It was a prospective study of Korean AR patients conducted from October 2013 to March 2015 at eight tertiary hospitals nationwide, and it was supported by the National Strategic Coordinating Center for Clinical Research under the Ministry of Health and Welfare.

Patients with AR symptoms who visited any of the eight medical centers that comprised the study sites were diagnosed with AR through history taking, endoscopic examination findings, skin prick test (SPT; Allergopharma, Reinbek, Germany) results, and serum laboratory analysis. Participants with at least one positive SPT result were considered to have AR. A total of 741 patients aged over 18 years were included in this study. The exclusion criteria were as follows: (1) patients with cardiovascular disease, serious disease involving other organs, chronic rhinosinusitis, nasal polyp, or psychiatric disorders such as depression; (2) those who had undergone sinus surgery; (3) those who could not express their opinions; and/or (4) those who could not answer the questionnaires properly.

### 2.2. Survey Questionnaires

The demographic data, AR symptom severity according to the ARIA classification (intermittent/persistent and mild/moderate–severe), perceived stress level (very low, low, moderate, or high), and disease-related QoL status on the Rhinoconjunctivitis Quality of Life Questionnaire (RQLQ) were measured using a survey questionnaire [17]. The questionnaire also included survey items on age, sex, monthly income, education, smoking status, high-risk drinking [18], number of siblings, body mass index (BMI), chronic conditions (asthma, hypertension, and diabetes), number of colds per year, global visual analogue scale (VAS) for AR, VAS for ocular discomfort, VAS for epiphora, perceived stress levels, and RQLQ.

The subjective degree of AR symptoms (global AR symptoms, ocular discomfort, and epiphora) was assessed using the VAS score ranging from 0 to 10 points. Perceived stress level was determined based on the response to the question [19,20] “How much stress do you have in your daily life during the past 1 year”, which was adopted from the Korea National Health and Nutrition Examination Survey (KNHANES) [21]. Patients were assigned scores from 0 to 3 according to their stress level: 0 = very low, 1 = low, 2 = moderate, and 3 = high. Participants were asked to grade the general stress they experienced in their daily lives, excluding that caused by AR symptoms. To measure disease-related QoL, we used the modified RQLQ from the validated Korean version, which contained 28 questions from 7 domains (activity limitations, sleep problems, nose symptoms, eye symptoms, non-nose/eye problems, practical problems, and emotional function). Participants were asked to report how bothered they were by their allergy symptoms during the previous week, and their scores ranged from 0 (not impaired at all) to 6 (severely impaired) [17,22]. The total RQLQ score was calculated as the mean of all 28 item scores, whereas the individual domain scores were the means of the scores of the items in those domains. Higher scores reflected worse disease-related QoL.

### 2.3. Skin Prick Test and Immunological Parameters

The SPT was performed on the medial sides of both forearms of the participants, using 13 common standardized allergen extracts (Allergopharma, Reinbek, Germany). The allergens were categorized into 7 groups: house dust mite (*Dermatophagoides pteronyssinus* (Dp) and *Dermatophagoides farinae* (Df)), animal danders (cat and dog), cockroaches (*Blattella germanica*), molds (*Alternaria alternata* and *Aspergillus fumigatus*), trees, grasses, and weeds (mugwort and ragweed). Histamine (1% histamine phosphate) and 0.9% saline were used as positive and negative controls, respectively. Skin sensitization (positive SPT) was defined as an average wheal diameter in response to any allergen greater than or equal to 3 mm. The numbers of categorized allergen groups with positive SPT results were collected. In addition, the serum total IgE levels and eosinophil counts of participants were measured via serum analysis.

### 2.4. Statistical Analyses

Patient characteristics are presented as means ± standard deviations for continuous variables and as frequencies (proportions) for categorical variables. The relationship between perceived stress level and RQLQ score was analyzed using univariable and multiple linear regression analyses. The assumptions in the linear regression model such as linear relationship, normality, and homoscedasticity were checked by residual plots. If homoscedasticity was violated, heteroscedasticity-consistent standard error estimators (HC3) were used to estimate the standard errors of model coefficients in regression models [23]. The relationship between perceived stress and RQLQ was estimated by adjusting the known confounders and covariates using the multiple regression model. All analyses were performed using SAS version 9.4 (SAS Institute, Inc., Cary, NC, USA), and a *p* value < 0.05 was considered statistically significant.

## 3. Results

### 3.1. General Association between Variables and RQLQ in AR Patients

**Figure 1:**
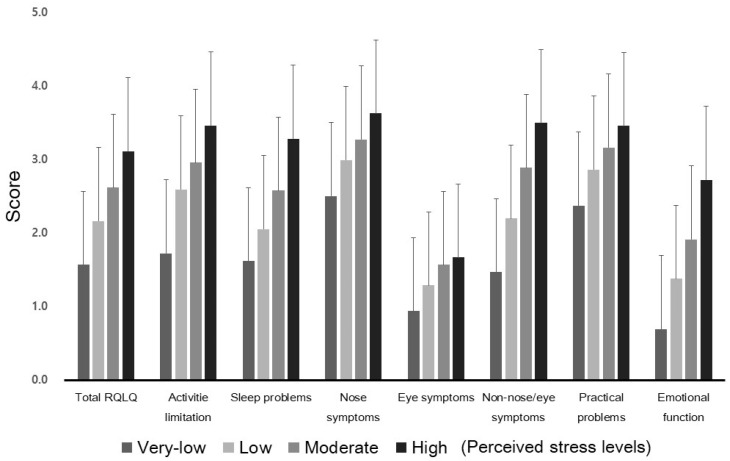
Total RQLQ score and domain scores according to perceived stress levels. The scores are shown as means with standard deviation. RQLQ: rhinoconjunctivitis quality of life questionnaire.

The general characteristics of the participants are presented in Table 1. The total RQLQ and score for each domain according to perceived stress level are shown in Figure 1. With univariate regression analysis, we analyzed the effect of the variables on total RQLQ (Table 2). Perceived stress levels were associated with a high total RQLQ score, which reflected poor disease-related QoL. Other variables that had a statistically significant relationship with the total RQLQ score were female sex, age, number of siblings, high-risk drinking, and number of colds per year (*p* < 0.05). As expected, the global VAS score, VAS score for ocular discomfort, VAS score for epiphora, persistent AR, and moderate–severe AR, all of which are closely related to AR symptom severity, were also associated with RQLQ score. Among the laboratory findings, eosinophil counts and the number of categorized allergen groups with positive SPT results were associated with an increased total RQLQ. The level of perceived stress was associated with the total RQLQ score. The total RQLQ score was elevated by 0.592 (95% confidence interval (CI), 0.311–0.873; *p* < 0.0001) when the stress level was low, 1.049 (95% CI, 0.755–1.343; *p* < 0.0001) when the stress level was moderate, and 1.543 (95% CI, 1.120–1.965; *p* = 0.0001) when the stress level was high. Female sex increased the total RQLQ score by 0.343 (95% CI, 0.138–0.547, *p* = 0.0011).

### 3.2. Association between Perceived Stress Levels and Total RQLQ

To analyze the effect of stress levels on total RQLQ score, the multiple regression model was adjusted for institute, age, sex, income, education, smoking status, high-risk drinking, number of siblings, BMI, asthma, hypertension, diabetes, number of colds per year, global VAS score, VAS score for ocular discomfort, VAS score for epiphora, AR severity based on ARIA classification, eosinophil count, total IgE, and number of positive allergen groups on the SPT (Table 3). Perceived stress level was associated with the total RQLQ score (Table 3). A low stress level increased the total RQLQ by 0.353 (reference, very-low-level-stress group; 95% CI, 0.099–0.606; *p* = 0.0065). The moderate and high stress levels increased the total RQLQ by 0.597 (95% CI, 0.327–0.867; *p* < 0.0001) and 1.210 (95% CI, 0.831–1.589; *p* < 0.0001), respectively.

### 3.3. Association between Perceived Stress Levels and Individual RQLQ Domain Scores

In the analyses of individual RQLQ domain scores, all domains except for practical problems (inconvenience of having to carry tissues, the need to rub the nose or eye, and the need to blow the nose repeatedly) and eye symptoms had significantly high RQLQ scores, especially in patients with a high level of perceived stress (Table 3). The domains for sleep problems and nasal symptoms were affected in the high-level-of-perceived-stress group compared with the very-low-level-stress group. The domains for activity limitations, non-nose/eye problems, and emotional function were affected in all the levels of the perceived stress group compared with the control group. The emotional function (2.081, 95% CI; 1.378–2.783; *p* < 0.0001) and activity limitation (1.633, 95% CI; 0.906–2.360; *p* < 0.0001) domains were most impaired, followed by the non-nose/eye problem domain (1.619, 95% CI; 1.015–2.223; *p* < 0.0001) and sleep disturbance domain (1.412; 95% CI; 0.667–2.157; *p* = 0.0002) in the high-perceived-stress group. The nasal symptom domain score showed a significant increment in the high perceived stress group (0.717; 95% CI; 0.083–1.350; *p* = 0.0267).

### 3.4. Contribution of Perceived Stress Levels to Total RQLQ

The adjusted R^2^ for the total RQLQ scores in AR participants was 0.5279, and the perceived stress levels contributed 4.08% in additional explanatory power to the total RQLQ score in AR patients (Table 4). The association between perceived stress level and total RQLQ score could be dependent on AR severity. Therefore, we additionally analyzed the interactions between AR severity and the total RQLQ; however, the relationship was not significant (*p* = 0.8176).

## 4. Discussion

In this study, stress level, which was easily indexed by simple questions, was significantly correlated with RQLQ score in AR patients. AR patients with a high level of perceived stress showed a lower disease-related QoL than those with a low level of perceived stress. These findings were consistent regardless of AR severity. To the best of our knowledge, this is the first report on the negative effect of perceived stress on disease-related QoL in AR patients.

The minimal clinically important difference of the RQLQ score has been found to be 0.5 on a scale ranging from 0 to 6 for the total RQLQ score [24]. In the current study, we demonstrated that perceived stress was associated with a significant elevation in RQLQ score of 0.592, even if the perceived stress level was low. Furthermore, a high perceived stress level was associated with a significant increase in the RQLQ score of 1.543.

There are very few previous studies on the relationship between stress and QoL in AR patients. In one study, 73.5% of patients with persistent AR had a positive result on the Kessler Psychological Distress Scale, which indicated a close association between persistent AR and psychological stress [15]. Patients with persistent AR who had a positive result on the Kessler Psychological Distress Scale and who were managed with antihistamines with imipramine demonstrated an improved QoL compared with the AR patients who were treated without imipramine. Kimata et al. [25] reported that stress-relieving activities such as listening to soothing music might decrease allergic responses by decreasing allergen-specific IgE levels. In our study, the multiple regression model adjusted for AR-related variables showed that 4.08% of the RQLQ score was explained by perceived stress.

We clearly demonstrated the effect of perceived stress on disease-related QoL in AR patients by classifying their perceived stress levels into four categories, using a simple question. We assessed the participants’ perceived stress levels based on their responses on a one-item questionnaire without using comprehensive stress measuring methods such as the Perceived Stress Scale (PSS) or the Daily Hassles Scale (DHS) [26]. This method of stratifying the stress level was simple and easy, and it enabled us to adequately assess the stress levels of AR patients with minimal effort. However, our use of such a simple categorization method might have resulted in the misclassification of the levels of perceived stress in the current study, although there was some evidence that the question we used had sufficient reliability for assessing stress levels [27].

There is accumulating evidence linking psychological stress to the expression of atopic disorders such as AR. Stress might have a direct effect and an important role in the onset and exacerbation of atopic disease through neuroendocrine and immunologic alterations [28,29,30,31]. Stress has been strongly associated with asthma incidence and hospitalization, use of asthma medication, AR, and atopic dermatitis in adults [29]. Moreover, there have been reports that maternal parenting and prenatal stress are related to the incidence of allergic diseases in children [32,33], suggesting the need for a multifaceted understanding of the relationship between stress and allergic diseases. As shown in our study, perceived stress was significantly associated with decreased disease-related QoL (high RQLQ score). Further study is needed to elucidate whether stress management leads to the improvement of QoL in AR patients. Studies exploring interventions such as medications, behavioral treatment, mental support, lifestyle modification, and adjuvant methods for relieving stress in the daily lives in AR patients receiving adequate medical treatment for allergic symptoms can further clarify the effect of stress on AR treatment.

We analyzed the total RQLQ score, which was the average of the sum of the scores of each item in seven domains and explored the possibility that a particular RQLQ domain might be more affected by perceived stress than others. Perceived stress led to impairments in both overall QoL as well as almost all the domains of rhinitis-related QoL such as nasal symptoms, non-nose/eye problems, sleep problems, activity limitations, and emotional function. Given that each domain reflected rhinitis symptom-related problems and the results were adjusted for AR severity, it was clear that perceived stress had an overall significant impact on rhinitis-related QoL. In addition to the medical and surgical treatment of allergic rhinitis, perceived stress in AR patients should be adequately assessed and actively managed to improve disease-related QoL.

The perceived stress of AR patients should be assessed in a feasible way at the clinic or bedside, and stress management should be considered as one of the strategies for managing AR. Based on the results of this study, a systematic approach for assessing and managing stress should be considered to be an important factor for improving the QoL of AR patients.

Although it provides evidence for the association between a high stress level and poor, disease-related QoL in AR patients, our study nonetheless has several limitations. First, while the simple question used to evaluate perceived stress in this study was very useful, further evaluation using various measures for evaluating stress might be more useful to study the association between stress and AR-related QoL. Second, as this was a cross-sectional study, the temporal link between perceived stress and disease-related QoL could not be assessed. Third, as we used self-reported data gathered using a questionnaire, there was a risk of imprecise recall and misclassification of perceived stress and QoL status.

## 5. Conclusions

AR patients with a high level of perceived stress showed a lower disease-related QoL compared with those with a low level of perceived stress. These findings were observed regardless of AR severity. These results highlight the need for clinicians to be aware of the stress status of AR patients. Controlling rhinitis symptoms is also important for improving the disease-related QoL of AR patients. Moreover, our results emphasize the importance of stress as a modifiable determinant of disease-related QoL. Assessment and active management of stress in AR patients may improve their disease-related QoL.

## Figures and Tables

**Table 1:** General characteristics of participants according to total RQLQ score and quartiles.

Variables		Total	Q1	Q2	Q3	Q4
RQLQ score	*n*	741	184	188	185	184
	Mean ± SD	2.3 ± 1.32				
	Median	2.29	0.64	1.75	2.82	3.93
Perceived stress level	Very low	87 (11.74)	41 (22.28)	22 (11.7)	15 (8.11)	9 (4.89)
(*n*, %)	Low	360 (48.58)	99 (53.8)	101 (53.72)	81 (43.78)	79 (42.93)
	Moderate	250 (33.74)	41 (22.28)	58 (30.85)	79 (42.7)	72 (39.13)
	High	44 (5.94)	3 (1.63)	7 (3.72)	10 (5.41)	24 (13.04)
Institute	1	59 (7.96)	17 (9.24)	12 (6.38)	18 (9.73)	12 (6.52)
(*n*, %)	2	95 (12.82)	31 (16.85)	20 (10.64)	25 (13.51)	19 (10.33)
	3	125 (16.87)	25 (13.59)	34 (18.09)	31 (16.76)	35 (19.02)
	4	59 (7.96)	19 (10.33)	22 (11.7)	9 (4.86)	9 (4.89)
	5	121 (16.33)	15 (8.15)	37 (19.68)	33 (17.84)	36 (19.57)
	6	95 (12.82)	24 (13.04)	23 (12.23)	23 (12.43)	25 (13.59)
	7	90 (12.15)	34 (18.48)	17 (9.04)	20 (10.81)	19 (10.33)
	8	97 (13.09)	19 (10.33)	23 (12.23)	26 (14.05)	29 (15.76)
Age	Mean ± SD	32.36 ± 12.72	34.78 ± 13.98	31.79 ± 13.27	31.64 ± 11.81	31.25 ± 11.45
Sex	Female	242 (32.66)	51 (27.72)	48 (25.53)	69 (37.3)	74 (40.22)
(*n*, %)	Male	499 (67.34)	133 (72.28)	140 (74.47)	116 (62.7)	110 (59.78)
Income	<2 M	94 (12.69)	20 (10.87)	22 (11.7)	26 (14.05)	26 (14.13)
(Korean won, KRW)	2 M–4 M	236 (31.85)	59 (32.07)	57 (30.32)	63 (34.05)	57 (30.98)
	4 M–6 M	187 (25.24)	49 (26.63)	45 (23.94)	51 (27.57)	42 (22.83)
	>6 M	224 (30.23)	56 (30.43)	64 (34.04)	45 (24.32)	59 (32.07)
Education	1 (lowest)	11 (1.48)	4 (2.17)	4 (2.13)	1 (0.54)	2 (1.09)
	2	38 (5.13)	9 (4.89)	10 (5.32)	12 (6.49)	7 (3.8)
	3	336 (45.34)	95 (51.63)	83 (44.15)	74 (40)	84 (45.65)
	4	304 (41.03)	62 (33.7)	69 (36.7)	90 (48.65)	83 (45.11)
	5	52 (7.02)	14 (7.61)	22 (11.7)	8 (4.32)	8 (4.35)
Number of siblings	Mean ± SD	1.89 ± 1.61	2.14 ± 1.76	1.86 ± 1.73	1.74 ± 1.42	1.83 ± 1.49
BMI (kg/m^2^)	Mean ± SD	23.34 ± 3.46	23.56 ± 3.44	23.35 ± 3.75	23.18 ± 3.36	23.27 ± 3.29
High-risk drinking	No	711 (95.95)	178 (96.74)	184 (97.87)	181 (97.84)	168 (91.3)
	Yes	30 (4.05)	6 (3.26)	4 (2.13)	4 (2.16)	16 (8.7)
Smoking status	Non-smoker	435 (58.7)	107 (58.15)	116 (61.7)	111 (60)	101 (54.89)
	Ex-smoker	142 (19.16)	43 (23.37)	32 (17.02)	28 (15.14)	39 (21.2)
	Smoker	164 (22.13)	34 (18.48)	40 (21.28)	46 (24.86)	44 (23.91)
Asthma	No	669 (90.28)	166 (90.22)	172 (91.49)	165 (89.19)	166 (90.22)
	Yes	72 (9.72)	18 (9.78)	16 (8.51)	20 (10.81)	18 (9.78)
Hypertension	No	699 (94.33)	169 (91.85)	175 (93.09)	181 (97.84)	174 (94.57)
	Yes	42 (5.67)	15 (8.15)	13 (6.91)	4 (2.16)	10 (5.43)
Diabetes	No	721 (97.3)	178 (96.74)	179 (95.21)	183 (98.92)	181 (98.37)
	Yes	20 (2.7)	6 (3.26)	9 (4.79)	2 (1.08)	3 (1.63)
Number of colds per year	Mean ± SD	2.87 ± 3.12	2.22 ± 2.24	3.03 ± 3.77	3.14 ± 3.14	3.1 ± 3.05
Global VAS score	Mean ± SD	6.84 ± 1.95	5.48 ± 2.14	6.51 ± 1.72	7.37 ± 1.37	8.03 ± 1.52
VAS score for eye discomfort	Mean ± SD	3.94 ± 3.22	1.99 ± 2.57	3.19 ± 2.93	4.52 ± 3.12	6.05 ± 2.75
VAS score for epiphora	Mean ± SD	2.08 ± 2.73	0.69 ± 1.3	1.32 ± 2.04	2.49 ± 2.84	3.83 ± 3.2
ARIA classification of AR	Intermittent AR	295 (39.81)	117 (63.59)	80 (42.55)	57 (30.81)	41 (22.28)
	Persistent AR	446 (60.19)	67 (36.41)	108 (57.45)	128 (69.19)	143 (77.72)
ARIA classification of AR	Mild	101 (13.63)	76 (41.3)	19 (10.11)	5 (2.7)	1 (0.54)
	Moderate–severe	640 (86.37)	108 (58.7)	169 (89.89)	180 (97.3)	183 (99.46)
Eosinophil count	*n*	662	159	170	167	166
(cells/μL)	Mean ± SD	266.84 ± 195.45	247.58 ± 184.18	243.59 ± 150.46	259.93 ± 193.02	316.05 ± 237.67
Total IgE	*n*	517	135	130	126	126
(kU/L)	Mean ± SD	315.75 ± 428.29	325.89 ± 421.32	287.19 ± 408.15	304.54 ± 405.86	345.57 ± 477.78
Number of positive SPT allergen groups	*n*	693	171	176	173	173
	Mean ± SD	2.59 ± 1.56	2.3 ± 1.38	2.56 ± 1.54	2.65 ± 1.7	2.84 ± 1.58

AR: allergic rhinitis; ARIA: Allergic Rhinitis and its Impact on Asthma; BMI: body mass index; M: million; Q: quartile; RQLQ: rhinoconjunctivitis quality of life questionnaire; SPT: skin prick test; VAS: visual analogue scale.

**Table 2:** Univariable regressions of the variables of the patients with AR for disease-related quality of life score (RQLQ score).

			HCSE Estimator			
		Estimate	SE	95% Confidence Interval		*p*-Value
Perceived stress level	Low	0.592	0.143	0.311	0.873	<0.0001 *
(reference = very low)	Moderate	1.049	0.150	0.755	1.343	<0.0001 *
	High	1.543	0.215	1.120	1.965	<0.0001 *
Institute	2	−0.115	0.220	−0.547	0.317	0.6013
(reference = 1)	3	0.195	0.208	−0.213	0.604	0.3484
	4	−0.329	0.241	−0.803	0.144	0.1724
	5	0.446	0.203	0.048	0.844	0.0281
	6	0.080	0.223	−0.359	0.518	0.7214
	7	−0.160	0.229	−0.611	0.290	0.4851
	8	0.333	0.221	−0.101	0.768	0.1322
Age		−0.009	0.004	−0.017	−0.001	0.0213 *
Female		0.343	0.104	0.138	0.547	0.0011 *
Income	2 M–4 M	−0.173	0.162	−0.492	0.146	0.2877
(reference = less than 2 M Korean won)	4 M–6 M	−0.218	0.165	−0.541	0.106	0.1871
	>6 M	−0.269	0.161	−0.585	0.047	0.095
Education	2	0.237	0.447	−0.640	1.114	0.5955
(reference = 1, lowest)	3	0.238	0.401	−0.550	1.025	0.5535
	4	0.476	0.402	−0.312	1.265	0.236
	5	−0.071	0.431	−0.916	0.775	0.8698
Number of siblings		−0.065	0.031	−0.126	−0.004	0.0367 *
BMI		−0.012	0.013	−0.038	0.015	0.3817
High-risk drinking		0.632	0.280	0.083	1.181	0.0241 *
Smoking status	Ex-smoker	−0.013	0.139	−0.287	0.261	0.9267
(reference = non-smoker)	Smoker	0.098	0.115	−0.127	0.324	0.3918
Asthma		0.046	0.161	−0.269	0.361	0.7747
Hypertension		−0.309	0.230	−0.760	0.142	0.1791
Diabetes		−0.395	0.347	−1.077	0.287	0.2555
Number of colds per year		0.046	0.016	0.016	0.077	0.003 *
Global VAS score		0.337	0.021	0.295	0.379	<0.0001 *
VAS score for eye discomfort		0.197	0.013	0.170	0.223	<0.0001 *
VAS score for epiphora		0.224	0.015	0.195	0.253	<0.0001 *
ARIA classification of AR, persistent		0.865	0.093	0.682	1.047	<0.0001 *
ARIA classification of AR, moderate-severe		1.673	0.090	1.496	1.850	<0.0001 *
Eosinophil count		0.001	0.0003	0.0005	0.002	0.0002 *
Total IgE		0.0001	0.0001	−0.0002	0.0004	0.5199
Number of positive SPT allergen groups		0.112	0.031	0.052	0.172	0.0003 *

AR: allergic rhinitis; ARIA: Allergic Rhinitis and its Impact on Asthma; BMI: body mass index; M: million; RQLQ: rhinoconjunctivitis quality of life questionnaire; SPT: skin prick test; VAS: visual analogue scale. * Univariate regression analyses, significance at *p* < 0.05. Heteroscedasticity-consistent standard error estimators, HC3, were used.

**Table 3:** The adjusted association between perceived stress level and RQLQ score.

			HCSE Estimator			
RQLQ Domain	Perceived Stress Level	Estimate	SE	95% Confidence Interval		*p*-Value
Total	Low	0.353	0.129	0.099	0.606	0.0065 *
	Moderate	0.597	0.138	0.327	0.867	<0.0001 *
	High	1.210	0.193	0.831	1.589	<0.0001 *
Activity limitations	Low	0.700	0.242	0.225	1.175	0.0039 *
	Moderate	0.920	0.256	0.418	1.422	0.0004 *
	High	1.633	0.370	0.906	2.360	<0.0001 *
Sleep problems	Low	0.025	0.250	−0.466	0.515	0.9217
	Moderate	0.411	0.263	−0.106	0.927	0.119
	High	1.412	0.379	0.667	2.157	0.0002 *
Nasal symptoms	Low	0.369	0.213	−0.050	0.788	0.0843
	Moderate	0.424	0.220	−0.009	0.857	0.0552
	High	0.717	0.322	0.083	1.350	0.0267 *
Eye symptoms	Low	0.020	0.137	−0.250	0.290	0.8869
	Moderate	0.019	0.148	−0.271	0.310	0.8967
	High	0.190	0.220	−0.243	0.623	0.3883
Non-nose/eye symptoms	Low	0.434	0.168	0.104	0.763	0.01 *
	Moderate	0.846	0.179	0.494	1.198	<0.0001 *
	High	1.619	0.307	1.015	2.223	<0.0001 *
Practical problems	Low	0.309	0.218	−0.119	0.737	0.157
	Moderate	0.363	0.229	−0.088	0.813	0.1141
	High	0.487	0.360	−0.220	1.194	0.1768
Emotional function	Low	0.546	0.156	0.241	0.852	0.0005 *
	Moderate	0.984	0.174	0.643	1.326	<0.0001 *
	High	2.081	0.357	1.378	2.783	<0.0001 *

RQLQ: rhinoconjunctivitis quality of life questionnaire. Multiple linear regression with heteroscedasticity-consistent standard errors (HSCE) for RQLQ score (*n* = 467, reference = very-low-level-stress group). * Linear regression analyses, significance at *p* < 0.05. The adjusted covariates in the model were institute, age, sex, income, education, smoking status, high-risk drinking, number of siblings, BMI, asthma, hypertension, diabetes, number of colds per year, global VAS, VAS for ocular discomfort, VAS for epiphora, AR severity based on ARIA classification, eosinophil count, total IgE, and positive allergen group number on SPT.

**Table 4:** Crude and adjusted R^2^ for RQLQ scores in AR participants.

	R-Square	Adjusted R^2^ *
Without perceived stress level	0.5234	0.4871
With perceived stress level	0.5644	0.5279

AR: allergic rhinitis; RQLQ: rhinoconjunctivitis quality of life questionnaire. * Stratified model with sociodemographics, chronic conditions, AR severity (according to ARIA classification and VAS), laboratory data (eosinophil counts, skin prick test results, and total IgE) and perceived stress level (very low, low, moderate, and high).

## Data Availability

All data are available upon request.
